# Breathlessness in a virtual world: An experimental paradigm testing how discrepancy between VR visual gradients and pedal resistance during stationary cycling affects breathlessness perception

**DOI:** 10.1371/journal.pone.0270721

**Published:** 2023-04-21

**Authors:** Sarah L. Finnegan, David J. Dearlove, Peter Morris, Daniel Freeman, Martin Sergeant, Stephen Taylor, Kyle T. S. Pattinson

**Affiliations:** 1 Wellcome Centre for Integrative Neuroimaging and Nuffield Division of Anaesthetics, Nuffield Department of Clinical Neurosciences, University of Oxford, Oxford, United Kingdom; 2 Oxford Centre for Diabetes, Endocrinology and Metabolism, University of Oxford, Oxford, United Kingdom; 3 Department of Psychiatry, University of Oxford, Oxford, United Kingdom; 4 MRC Weatherall Institute of Molecular Medicine, University of Oxford, Oxford, United Kingdom; Sheffield Hallam University, UNITED KINGDOM

## Abstract

**Introduction:**

The sensation of breathlessness is often attributed to perturbations in cardio-pulmonary physiology, leading to changes in afferent signals. New evidence suggests that these signals are interpreted in the light of prior "expectations". A misalignment between afferent signals and expectations may underly unexplained breathlessness. Using a novel immersive virtual reality (VR) exercise paradigm, we investigated whether manipulating an individual’s expectation of effort (determined by a virtual hill gradient) may alter their perception of breathlessness, independent from actual effort (the physical effort of cycling).

**Methods:**

Nineteen healthy volunteers completed a single experimental session where they exercised on a cycle ergometer while wearing a VR headset. We created an immersive virtual cycle ride where participants climbed up 100 m hills with virtual gradients of 4%, 6%, 8%, 10% and 12%. Each virtual hill gradient was completed twice: once with a 4% cycling ergometer resistance and once with a 6% resistance, allowing us to dissociate expected effort (virtual hill gradient) from actual effort (power). At the end of each hill, participants reported their perceived breathlessness. Linear mixed effects models were used to examine the independent contribution of actual effort and expected effort to ratings of breathlessness (0–10 scale).

**Results:**

Expectation of effort (effect estimate ± std. error, 0.63 ± 0.11, *P* < 0.001) and actual effort (0.81 ± 0.21, *P* < 0.001) independently explained subjective ratings of breathlessness, with comparable contributions of 19% and 18%, respectively. Additionally, we found that effort expectation accounted for 6% of participants’ power and was a significant, independent predictor (0.09 ± 0.03; *P* = 0.001).

**Conclusions:**

An individuals’ expectation of effort is equally important for forming perceptions of breathlessness as the actual effort required to cycle. A new VR paradigm enables this to be experimentally studied and could be used to re-align breathlessness and enhance training programmes.

## Introduction

Breathlessness is a complex perception, in which the brain is now recognised as an active contributor. Rather than passively transmitting sets of afferent signals, new evidence suggests that the brain interprets incoming signals based on a set of held “expectations” [1–3]. Because perceptions, including those of breathlessness are generated in the brain, a disconnect between the lungs and perception can be explained as a mismatch between the senses [1, 4]. Such a disconnect may explain why breathlessness is often difficult to treat, with many people, such as those living with chronic lung or cardiac disease remaining symptomatic despite maximal medical therapy [5–7]. For these people, the sensation of breathlessness may not match the physical status of the cardiorespiratory system. Indeed, sensory mismatches resulting in breathlessness can be generated experimentally via engendered expectations to placebo and nocebo cues [8]. Conversely, treatments drawing on exposure based cognitive therapies, which focus on changing sensory and emotional expectations, including those relating to anxiety and mood, appear to be most successful in providing symptomatic relief from chronic breathlessness [9, 10].

Current evidence highlights that an individual’s expectations about breathlessness and exercise may influence their subjective experiences. For example, an individual who realises they have forgotten their inhaler may suddenly experience feelings of shortness of breath. However, given that breathing contains both conscious and unconscious elements, is emotionally complex and difficult to articulate, quantifying this relationship is challenging.

Recent work has built on well-established embodiment illusions, such as the rubber-hand illusion [11], in which participants “feel” the rubber hand as their own to show how visual and somatosensory cues can be used to “trick” perceptions of bodily status. Using virtual avatars, manipulating the avatar’s respiratory phase directly altered feelings of participant self-location, breathing agency and tidal volume [12]. The affective impact of breathlessness was also attenuated by the presence of a virtual avatars breathing, particularly when asynchronous with a participants own breathing [13]. Manipulating this sense of agency is not restricted to passive situations or to external cues. While exercising under hypnosis, ratings of perceived exertion have been shown to reflect the suggested, rather than actual work effort [14]. Similarly, during imagined hand-grip exercises, individuals with high hypnotizability perceived their rates of exertion as higher than those with low hypnotizability [15].

One key aspect that drives expectation is sensory immersion, which now, rather than relying on hypnosis or carefully contrived illusions, is immediately available under full control via Virtual Reality (VR) technology. As a result, VR technology is emerging as a powerful tool within healthcare and research settings, allowing therapeutically relevant situations to be created, repeated and manipulated with a consistency impossible to create in real life [16, 17]. The situations created by VR are immediately available (and readily ended if participants become uncomfortable), facilitating patient access and engagement in a safe space. Meanwhile, researchers can create “real-world” environments whilst maintaining carefully controlled experimental parameters. Thus, VR has the potential to disentangle how prior expectations contribute to breathlessness.

Virtual reality has already been used therapeutically to reduce complex regional pain [17], improve the acceptance and embodiment of prosthetic limbs [18] and facilitate recovery post-stroke [18]. However, VR interventions for chronic breathlessness are in their infancy. Gamification of physical exercise has been positively received [19, 20], with participants reporting higher enjoyment and self-efficacy compared to traditional static cycling. Associated feelings relating to accomplishment may also address mood and affect, which have been highlighted as key factors in the breathlessness experience [5, 21]. A recent systematic review which found moderate effects of VR-exercise (including full body work out, jogging and balance training) on breathlessness, and weak effects on heart rate and oxygen saturation over traditional exercise, highlighted the limited number of studies and lack of standardization of methodology. Furthermore, none of the virtual gaming interventions were immersive, i.e. using a reality headset. Therefore, it remains unknown whether perceptions of breathlessness can be independently manipulated via a VR exercise paradigm.

The primary aim of this study was to use a novel immersive VR exercise paradigm to determine whether subjective sensations of breathlessness could be manipulated independently of physical effort by modulating an individual’s expectation of effort. Secondary study aims were to determine the effect of modulating an individual’s expectation of effort on power produced during cycling exercise and to determine participants’ immersion within the VR exercise paradigm.

## Methods and materials

### Research design and participants

Nineteen healthy adult participants (10 female, median age 20 years; range 19–41 years) were recruited to a random order controlled, parallel design study. The study was granted approval by University of Oxford, Medical Sciences Interdivisional Research Ethics Committee (Ref: R68447/RE001). Participants responding to study adverts were screened via telephone to determine fulfilment of inclusion criteria, before being invited to attend a single one-hour study visit at the Department of Physiology, Anatomy and Genetics, University of Oxford. Study inclusion criteria were adults aged 18–65 y with no cardiac or metabolic disease, neurological impairment, psychiatric disorder or prescription/non-prescription drug dependency. Exclusion criteria were significant deviation from study protocols or illness/injury that would impair exercise capacity or perceptions of breathlessness during exercise. Written informed consent was obtained from all participants before enrolment and all procedures were conducted in accordance with the Declaration of Helsinki. Participants were randomly allocated to one of two exercise protocols (discussed below) based on order of enrolment using a counterbalanced matrix. Participants were informed that they would be required to cycle on a static bicycle while wearing a virtual reality headset and report their breathlessness and that they would complete a series of questionnaires. Participants were not informed prior to the experiment that the virtual slope would at times differ from the physical work effort.

### Study visit protocol

#### Self-report questionnaires

To assess whether baseline assessments of anxiety influenced either perceived breathlessness or modulated the interaction between VR hill gradient and physical effort, participants completed the state component of the State-Trait Anxiety Inventory (STAI) [22] before commencing exercise. The STAI questionnaire is a commonly used self-report questionnaire designed to measure anxiety consisting of 40 items on a 4-point Likert scale (1. Not at all, 2. Somewhat, 3. Moderately so, 4. Very much so). The state component assesses anxiety in the present moment rather than more generally and consists of 20 of the 40 items. To assess levels of immersion within the virtual environment we collected scores on the Presence Questionnaire (PQ) [23]. The PQ is a validated 19 item questionnaire (or 24 if the environment included haptics and sound—which for this study were not relevant). Each question on the presence questionnaire is scored out of a maximum of 7 points on a Likert scale, with one being “not at all” and seven being “very much”. Including this questionnaire captures information relating to the success of the software to generate an immersive experience. Breathlessness was measured using a simple visual analogue scale, further details are included below within cycling protocol. Questionnaires were scored according to their respective manuals.

#### Anthropometric assessments

Participant stature (Seca 213 Stadiometer, Seca GmbH, Birmingham, UK) and body mass (BF508 scales, Omron, Kyoto, Japan) were assessed upon arrival to the laboratory. BMI (kg/m^2^) was subsequently calculated and given the known associations between adiposity and exercise capacity [24], included as a covariate in regression models investigating the effect of effort expectation on subjective sensations of breathlessness and power during cycling exercise.

#### Cycling protocol

**Fig 1:**
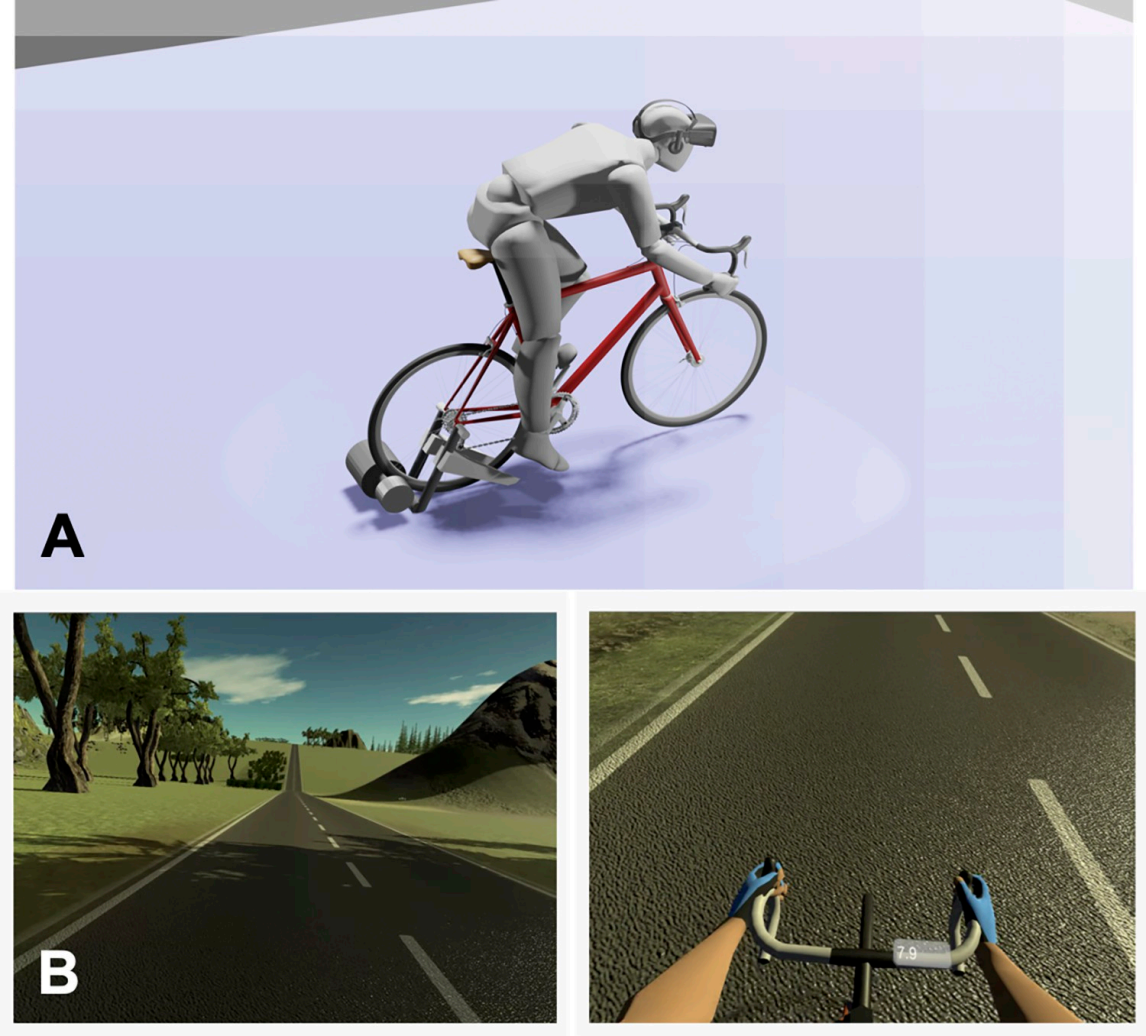
A. Image of the bicycle ergometer set-up and B. (top) the VR environment (bottom) virtual hands and speedometer.

Exercise was performed on a bicycle attached to a Tacx Flow Smart turbo trainer (Garmin Ltd., Kansas, US) that recorded power output and communicated directly with the VR software (Fig 1A). The turbo trainer was calibrated following manufacturer guidelines prior to each exercise session and tyre pressure was maintained at 90 Psi, as per manufacturer guidelines. Participants adjusted the bicycle saddle height according to personal preference, air conditioning was fixed at 16°C and a fan (ProBreeze 20 inch floor fan, Pro Breeze, London, UK) was positioned in front of participants and set to speed 2.

**Fig 2:**
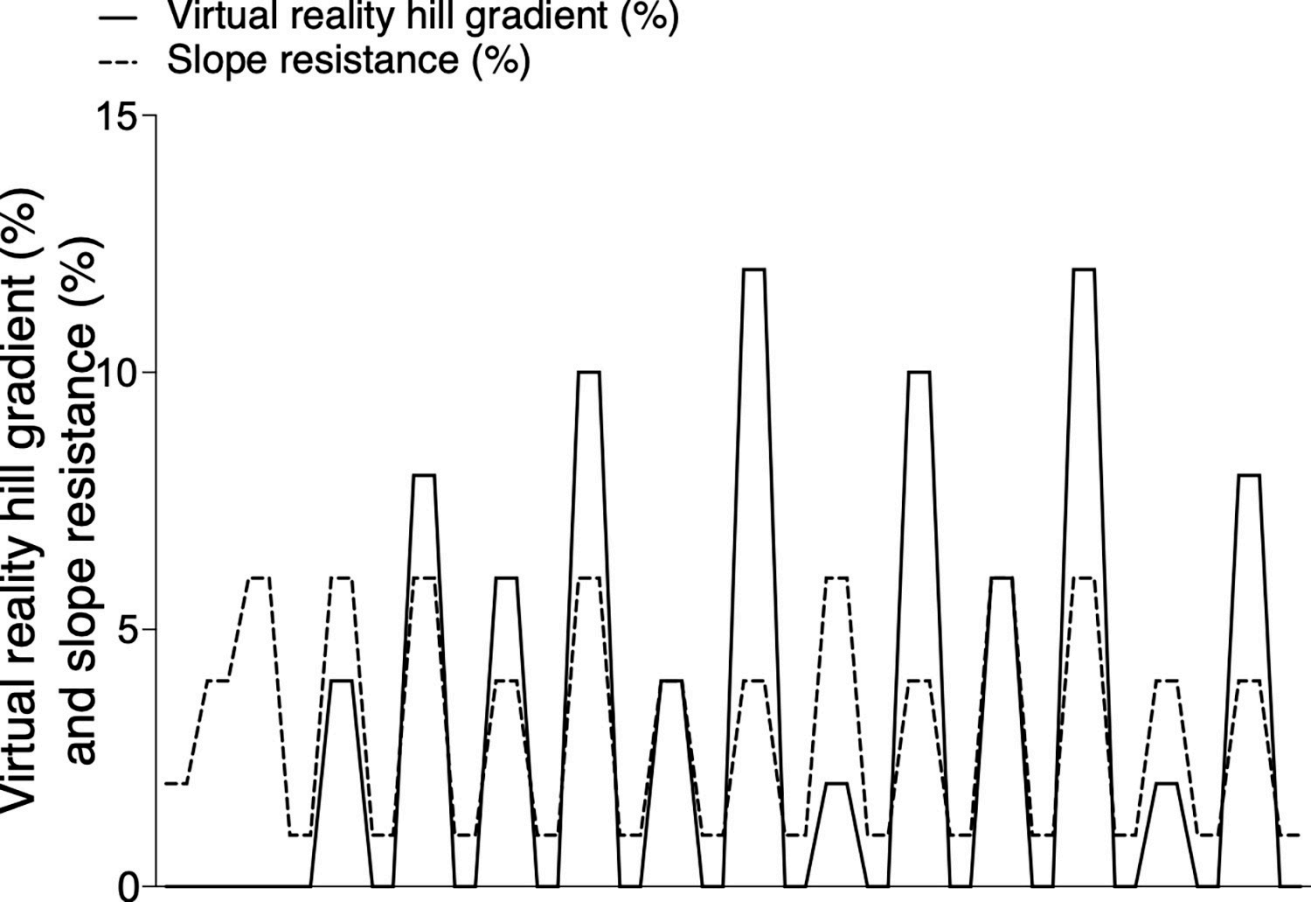
Illustration of exercise protocol where slope resistance (cycling ergometer resistance) was dissociated from the observed VR hill gradient.

Participants completed two exercise blocks of cycling in a virtual world with a rolling terrain (Fig 1B), where the power was independently manipulated from the observed VR hill gradient. The first exercise block was used to familiarise participants to slope resistances and VR hill gradients. Participants then rested for an average of 4 minutes 40 seconds before starting the second exercise block, informing the experimenter when they were ready to begin. Only data from the second exercise block were included in the final analyses. In each exercise block, participants warmed-up for 300 m (3 x 100 m with 2%, 4% and 6% slope resistances, respectively) before completing an undulating course containing 12, 100 m long hills with observed gradients of 4%, 6%, 8%, 10% or 12% (Fig 2). Participants completed each hill gradient twice, once with a slope resistance of 4% and once with a slope resistance of 6% (Fig 2). Slope resistances were determined by the pressure applied by the turbo trainer to the rear tyre and designed to mimic the resistances experienced (e.g., from air, gravity and friction) whilst cycling up 4% and 6% gradients. Power was not fixed, and participants cycled at a self-selected effort. After each 100 m hill, a virtual prompt appeared asking participants to report how breathless they felt on a scale of 1–10 (asked as: “on a scale of 1–10, 1 being not at all breathless and 10 being maximum breathlessness, how breathless do you feel”). Participants’ verbal report was recorded automatically by the computer, and this was verified by a study investigator. We designed 2 exercise blocks that differed in the order VR hill and slope resistance combinations were experienced (see S1 Table in S1 File for exercise block designs). Participants were randomised in the order they undertook the exercise blocks, such that approximately half completed block design 1 first (familiarisation) and block design 2 second (experimental) and vice versa. This enabled us to account for any effects of cumulative exercise fatigue on perceptions of exercise exertion and power.

#### Software

The VR software was written in Unity (version 2019.2.17), using the Unity packages VRTK (version 3.3.0) for integration with VR and Advanced ANT+ (version 1.041) for communication with the turbo trainer (or any fitness equipment using the ANT+ protocol). In addition, an Arduino UNO microcontroller was integrated into the system, to allow analog/digital inputs. The virtual world was viewed using an Oculus Rift headset. We used two commercially available libraries from the Unity Asset Store; Advanced ANT+:

https://assetstore.unity.com/packages/tools/network/advanced-ant-71980#description and Bicycle Power Simulator:

https://assetstore.unity.com/packages/tools/physics/bicycle-power-simulator-71573#description.

Since these are commercial packages, we provide a compiled version of the complete software for download at https://zenodo.org/deposit/6706457.

#### Analysis

All variables were centred and scaled in R prior to analysis. To assess whether slope resistance (4% and 6%) affected participants’ physical effort (Watts), a 2-way repeated-measures ANOVA was performed. To determine the effect of actual effort and effort expectations on breathlessness, and to determine the effect of effort expectation on actual effort, linear mixed-effects models were created in RStudio (Version 1.3.1093) using the lme4 package [25]. For both models, a random intercept was fitted for each participant. This accounted for differences within cardiorespiratory fitness by allowing each participant to act as their own statistical baseline. Final models (combinations of predictor variables) were selected by Akaike Information Criterion (AIC) by backwards elimination using the statistical package “stats” within R [26]. Reported effect estimates for each fixed effect represent the change in the response variable (e.g., perception of breathlessness) when a fixed effect variable changes by one unit, while other fixed effect variables are held constant at their mean value. VR Full model summaries and terms are presented in S1 File.

## Results

### Participant characteristics

Nineteen participants completed the trial and were included in final analyses (see Table 1 for participant characteristics). Participants were on average below clinical thresholds for anxiety (25 (10), typically defined as a score of 40 or more [27] (Table 1).

**Table 1:** Demographic information.

N = 19	Score
**Age (median years/range)**	20 (19–41)
**BMI (kg.m**^**-2**^ **± SD)**	22 ± 2
**Stature (cm ± SD)**	175 ± 8
**Body mass (kg ± SD)**	67.33 ± 9.24
**Gender–Female**	11

### Exercise time to completion

The mean ± SD power for the 3 x 100 m warm-up segments at 2%, 4% and 6% slope resistances were 68 ± 17 W, 143 ± 28 W and 177 ± 40 W, respectively. The mean time taken to complete the familiarisation block was 7 minutes and 12 seconds (SD 1 minute). The mean rest period was 4 minutes and 40 seconds (SD 1 minute 48 seconds). The mean time taken to complete cycling block two was 6 minutes and 39 seconds (SD 1 minute 10 seconds). The familiarisation block and block 2 was comparable (*P* > 0.05).

### Association between slope resistance and power

**Fig 3:**
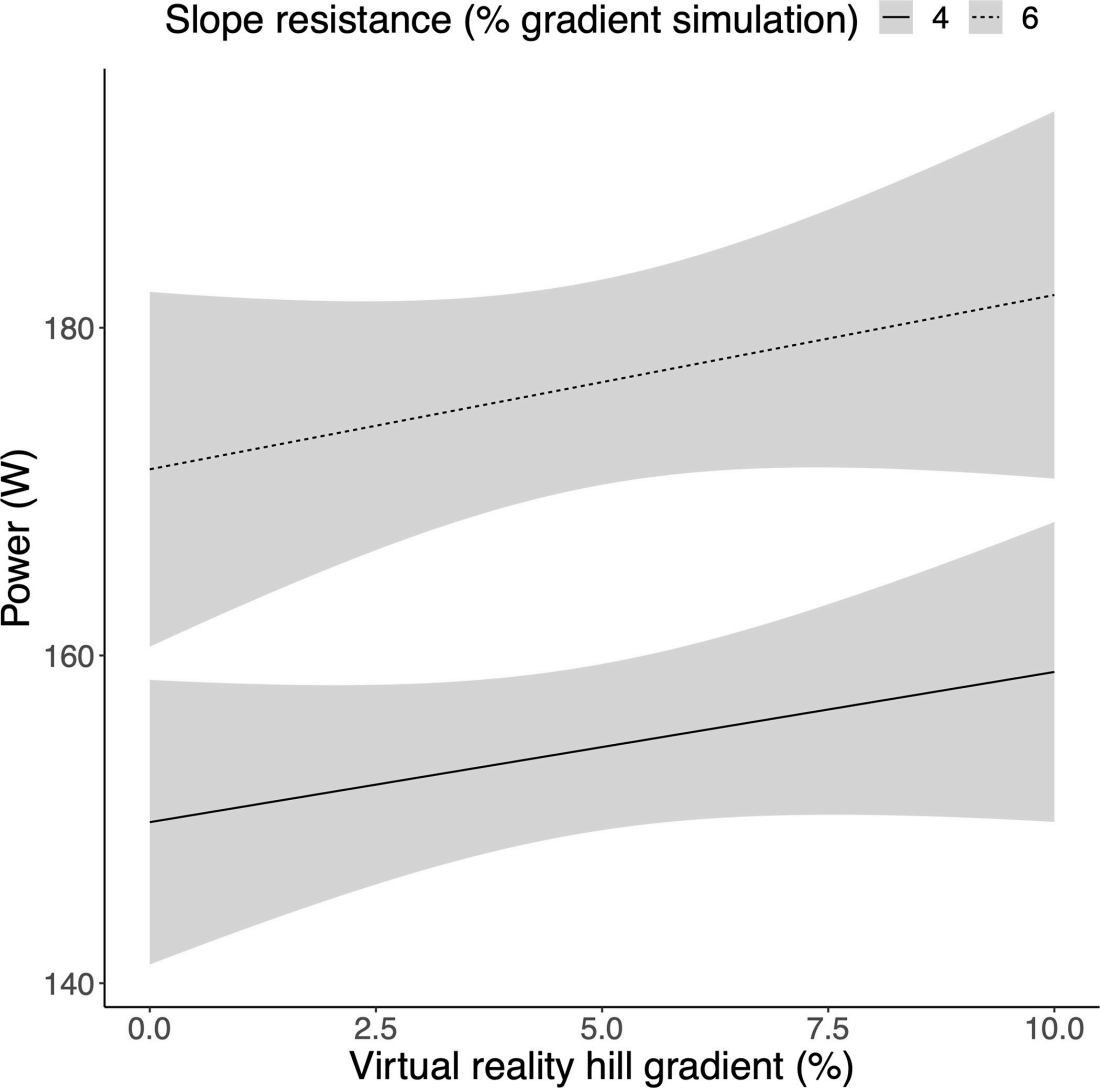
Slope resistance is a significant predictor of power. Mean power (W) was greater across all levels of VR hill gradient in the 6% vs. 4% slope resistance blocks. Shaded areas represent 95% CIs.

To test the hypothesis that participants exerted greater physical output during the 6% vs. 4% slope resistance blocks, a 2-way ANOVA was performed where power (W) was explained as a function of slope resistance, VR hill gradient and their interaction (see S2 Table in S1 File for full ANOVA results). There was a significant main effect of slope resistance (*P* < 0.001; Fig 3), demonstrating that participants produced more power in the 6% vs. 4% blocks. This relationship was consistent across all levels of VR hill gradient, as evidenced by a non-significant interaction effect (*P* > 0.05).

### Virtual reality effort is an independent predictor of breathlessness

**Fig 4:**
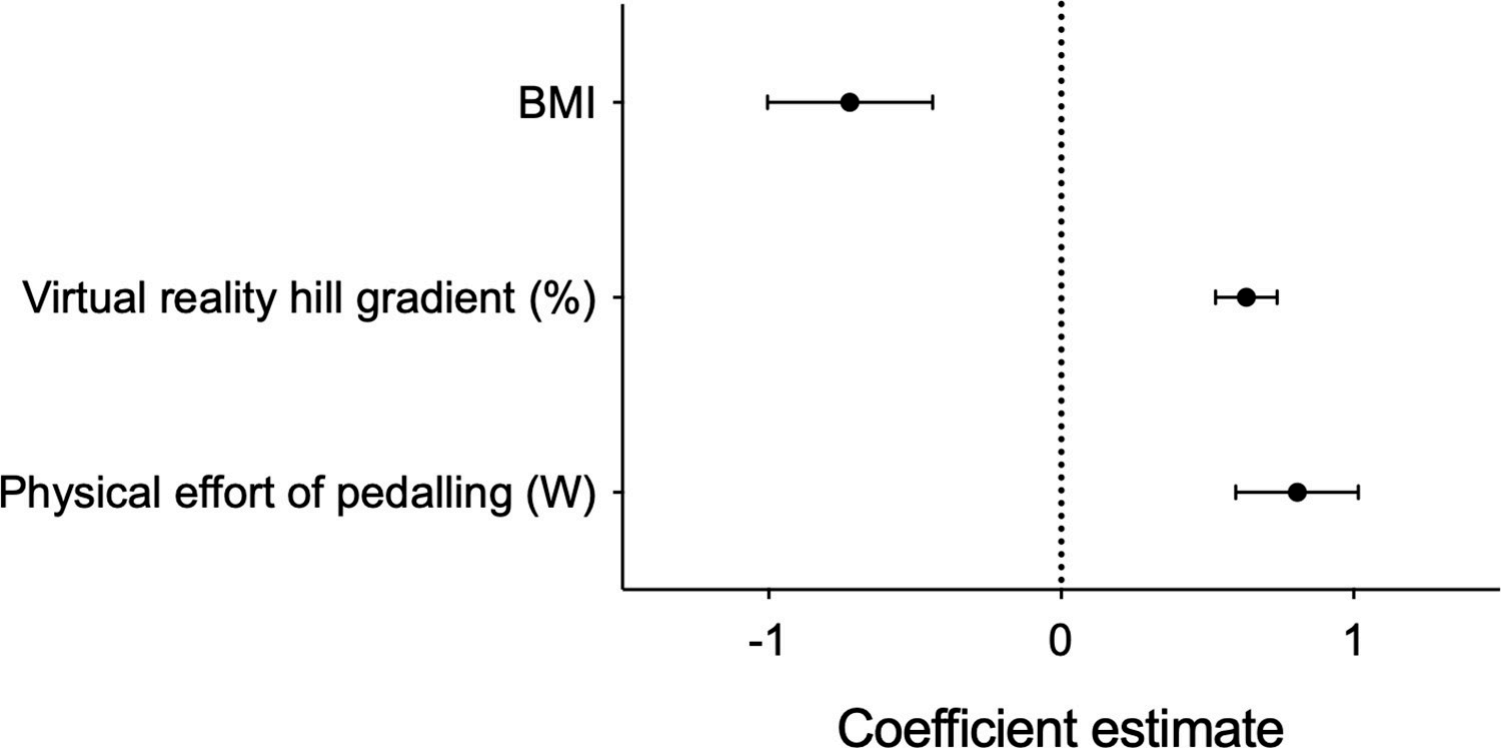
The power (actual effort), virtual hill gradient (expected effort), and BMI are independent predictors of an individual’s breathlessness.

To determine whether an individual’s expectation of effort was independently associated with breathlessness, a mixed effects regression model was created. Here, subjective breathlessness rating (1–10 Likert scale) was explained as a function of the power (W), VR hill gradient, state anxiety level before undertaking exercise (STAI questionnaire), age, sex, and BMI, with a random participant effect included. Following model optimisation through backwards elimination of non-significant model terms (see S3 Table in S1 File for the full and optimised models), power (W) (Effect estimate: 0.81 ± 0.21, 95% CI [0.39, 1.22], *P* < 0.001, R^2^ = 0.175) and virtual hill gradient (Effect estimate: 0.63 ± 0.11, 95% CI [0.43, 0.84], *P* < 0.001, R^2^ = 0.192) were positively associated with subjective breathlessness ratings, whilst BMI was significantly negatively associated (Effect estimate: -0.72 ± 0.28, 95% CI [-1.28, -0.17], *P* = 0.02, R^2^ = 0.198) (Fig 4). Variance inflation was low (score < 1.2) for all model terms, suggesting minimal collinearity.

### Effort expectation in VR modulates actual physical effort

**Fig 5:**
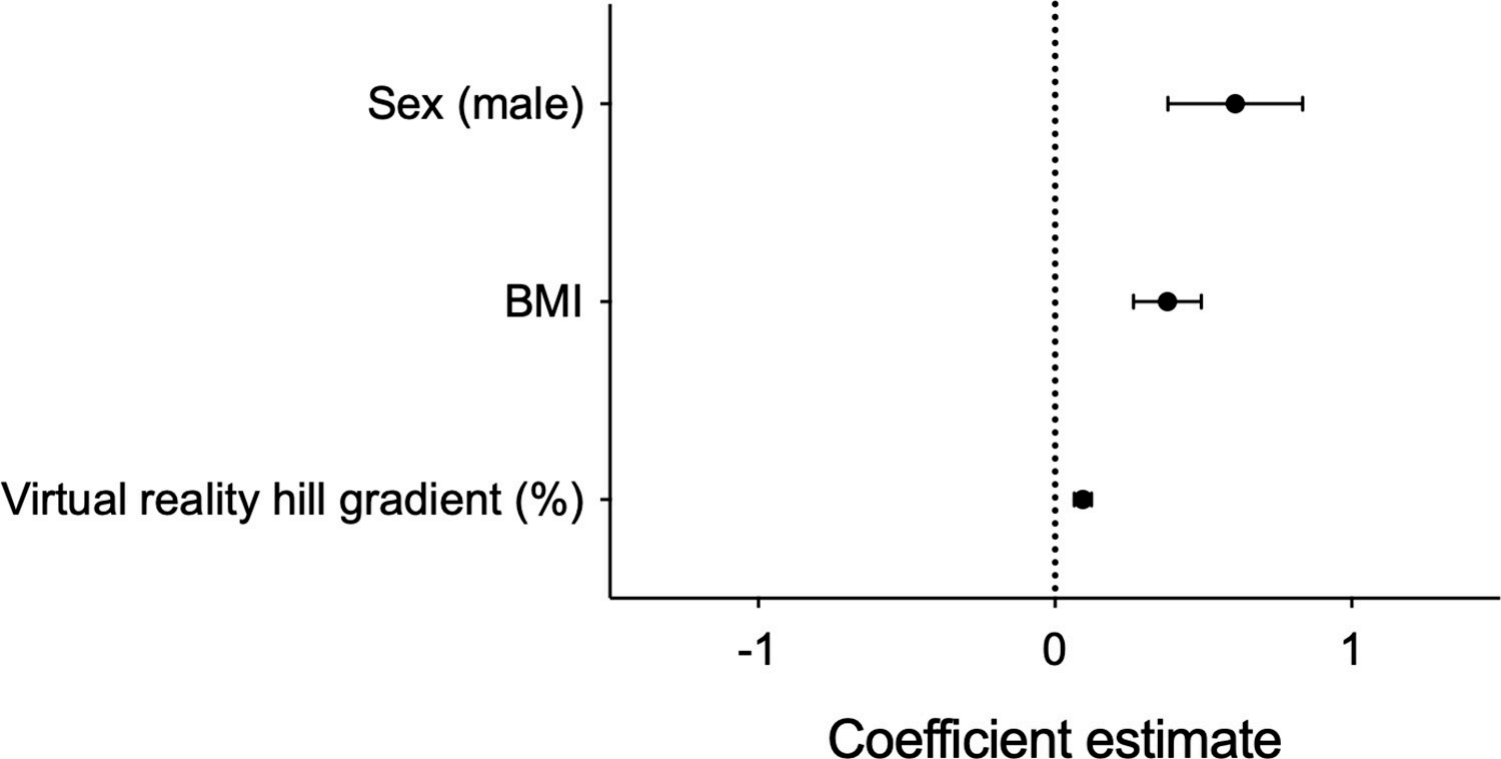
Virtual reality hill gradient, BMI, sex (male) are independent predictors of physical effort (i.e., the power).

Finally, we sought to determine whether effort expectation would influence participants’ actual effort. We created a second mixed effects model where the power (W) was explained as a function of virtual hill gradient, BMI, age, and sex, with a random participant effect included. Following model optimisation through backwards elimination of non-significant model terms (see S4 Table in S1 File for the full and optimised models), virtual hill gradient (Effect estimate: 0.09 ± 0.03, 95% CI [0.04, 0.15], *p* = 0.001, R^2^ = 0.060), BMI (Effect estimate: 0.38 ± 0.11, 95% CI [0.15, 0.60], *p* = 0.004, R^2^ = 0.518), and sex (male) (Effect estimate: 0.61 ± 0.23, 95% CI [0.16, 1.06], *p* = 0.02, R^2^ = 0.411) were positively associated with the power (Fig 5). Variance inflation was low (1.0) for all model terms, suggesting minimal collinearity.

### Immersion in the VR cycling world

Participants reported a median score of 5.25 (IQR 1). Highest item scores were recorded for rapid adjustment to the virtual world and for the quality of display. Lowest scores were recorded for the item examining how consistent participants felt their experiences in the virtual world were compared to the real-world (Table 2).

**Table 2:** Item and total scores for the presence questionnaire (PQ).

Question	Median score out of 7 (IQR)
**How natural was the mechanism that controlled movement through the environment**	4.5 (1.5)
**How compelling was your sense of objects moving through space?**	5 (2)
**How much did your experiences in the virtual environment seem consistent with your real-world experiences?**	4 (1)
**How involved were you in the virtual environment experience**	5.5 (1)
**How quickly did you adjust to the virtual environment experience**	7 (1.5)
**How much did the visual display quality interfere or distract you from performing assigned tasks of required activities?** (reverse scored)	6 (2.5)
**Total average score**	5.25 (1)

## Discussion

### Key findings

The primary aim of this study was to determine whether subjective sensations of breathlessness could be manipulated independently of physical effort. In this study, we used a novel VR cycling paradigm to dissociate actual effort (the power required to pedal) from an individual’s effort expectation (observed VR hill gradients) and in doing so determined the contribution of effort expectation to the sensation of breathlessness. We found that approximately 19% of the breathlessness experienced was explained by the VR hill gradient observed by participants, whereas approximately 18% was explained by the power required by participants to pedal. Additionally, we showed that participants cycled with more power when they observed a steeper virtual slope, despite ergometer resistance being kept constant, with the observed VR hill gradient accounting for 6% of the power produced by participants. These findings demonstrate that an individual’s expectations of effort can independently modulate subjective perceptions, including breathlessness, and influence physical effort. This work is a clear first step towards realigning expectation with sensory inputs, a key aspect of many exposure based cognitive behavioural therapies.

### Examining the contribution of expectations in chronic breathlessness

A growing body of evidence [1–4, 8, 10, 28–31] now suggests that breathlessness, far from simply arising as a product of peripheral inputs, includes steps of active interpretation and comparison with previously held expectations. A good example of this can be found within the placebo/nocebo effect demonstrated by one study of healthy volunteers in which, using a conditioning paradigm, a harmless odour was paired with induced breathlessness. Subsequently, the odour alone was shown to lead to breathlessness and associated brain activity patterns despite the absence of afferent respiratory input. The strong influence of expectation and its relationship tied to emotions of mood and anxiety may explain why breathlessness severity is often poorly explained by objective clinical measures [32]. This is particularly relevant to instances of unexplained breathlessness such as can be observed in asthma or panic disorder, where breathlessness fails to match physiological assessments of cardiopulmonary health. In this study we demonstrated that as the observed VR hill gradient increased, participants’ reported breathlessness was also increased, independent of the physical effort applied to pedalling the bike. This highlights that an individual’s prior expectations may influence their subjective experiences of exertion, including how breathless they feel in response to a given exercise intensity. This work is in line with previous studies showing, in cases of chronic breathlessness, that improvements in breathlessness over pulmonary rehabilitation are associated with a reappraisal of the sensory experience, i.e., changes to expectations [9] rather than increased fitness. These changes are mirrored by changes to brain activity within key areas relating to attention and learned sensory and emotional expectations such as the cingulate cortex, insula and angular gyrus [9]. However, determining the contribution of ‘expectation’ to the subjective human exercise experience is problematic, particularly where beliefs or expectations are difficult to articulate. Therefore, paradigms in which expectation can be uncoupled in a systematic manner from power output may now provide a tool with which to unpick this relationship.

### Physical effort is influenced by virtual slope

In the real world, road inclinations are met with proportional increases in resistance due to gravitational force and because of learning about our physical environment, our expectation is that we must apply greater power to maintain our speed. In this experiment we observed a positive association between the virtual slope gradient and the pedalling power applied by participants, independent of ergometer resistance. Put simply, as the virtual slope appeared to become steeper, participants cycled harder as if in anticipation of having to apply more work, suggesting participants were strongly immersed within the environment. Similar to the rubber hand illusion, in which participants “felt” the rubber hand to be their own, the VR environment created within this study was sufficiently immersive to directly impact participant’s behaviour. To the authors’ knowledge, this is the first study to demonstrate that manipulating expectations within a virtual environment influences an individual’s physical effort. While, our study did not investigate exercise performance, this finding nevertheless suggests that manipulating sensory or environmental cues could be used to increase physical capacity in rehabilitation settings (e.g., cardiac recovery or pulmonary rehabilitation) and more broadly, training of the general population. This could be achieved via two mechanisms. In the first, participants could be shown a steeper virtual hill but experience a consistent low resistance to build a sense of accomplishment, resetting expectations and overcoming the spiral of avoidance, fear and deconditioning common to chronic breathlessness [33]. In a second mechanism the virtual slope could be maintained at a low gradient, while applying an increased resistance. This would build physical strength while avoiding the spiral of fear and avoidance. Both approaches draw on theories of “treat the lungs, fool the brain and appease the mind” [10] in which the “top-down” brain outputs and “bottom up” physical signalling must remain in balance to avoid unexplained breathlessness.

### Limitations

The purpose of this study was to demonstrate that subjective breathlessness ratings could be manipulated independently from physical work effect. Given the novelty of the design and lack of established evidence within the field to draw upon we have identified a number of key limitations that future studies will work to address.

In terms of hardware, while VR headsets provide a fully immersive experience, the headset generates a noticeable amount of heat, which paired with physical exertion can quickly become uncomfortable for the participant. Although this does not appear to have negatively affected immersion in this study, future studies using longer exercise durations may investigate other display options such a wide screen monitors, immersive rooms or enlist newer headsets with built in cooling systems. Within a future study we would also capture information regarding temperature and humidity, which was not recorded in this study, although air-conditioning temperature and fan speed were controlled for. In this single session we assessed the extent to which the illusion was maintained across a wide range of virtual slopes. However, the Tacx turbo trainer was only capable of simulating gradients of 0% to 6%. Consequently, the range of physical slopes did not align with the range of virtual slopes. Future studies would improve statistical power by limiting the range of virtual slopes under comparison, extend either the number of sessions or time over which data was collected and assess the ideal ratio of timing between rest/reset blocks and exertion blocks. Additional psychological and physiological characterisation would also help to answer some outstanding questions. Further physiological assessments, quantified by cardiopulmonary exercise testing, including a baseline assessment of cardiorespiratory fitness would help to answer questions regarding whether the virtual environment simply changed perceptions of work effort on a cognitive level or whether an individual’s physiology was also affected. Previous work using hypnosis has demonstrated that manipulating expectation activates cortical structures involved in the cardiopulmonary response to exercise, resulting in increased blood pressure and heart rate [14]. While in terms of psychological characterisation the presence questionnaire is somewhat limited by a lack of validated thresholds. While in this study we did not identify a relationship between baseline anxiety and self reported breathlessness, our population were in general healthy non-anxious adults. Further assessment of anxiety and interoceptive sensitivity and its relationship with manipulability of expectation may also be of interest. Studying more highly anxious individuals would be worthwhile as we might predict that the effects of VR manipulation may be even greater. Finally, this study has provided initial evidence for the manipulation of breathlessness expectations within a healthy population. Further work would extend this paradigm to assess utility in clinical populations and in a larger, more heterogenous population.

### Future directions

Realigning expectations with sensory inputs and challenging aberrant thought processes is a key foundation of many cognitive behavioural therapies. Compelling evidence highlights how pulmonary rehabilitation, the current most effective treatment for chronic breathlessness in COPD, has little effect on lung function but significantly improves feelings of breathlessness via changes in brain processes [9]. While a number of brain-targeted therapeutic approaches, including pharmacological [34, 35], direct brain stimulation [36] and cognitive [37] have been explored with varying degrees of success, VR offers another approach in which sensory input may be realigned with expectation in an example of “fool the brain, treat the lungs” [10]. The high entertainment value of immersive VR, good immersion as demonstrated by scores on the presence questionnaire, and increasing affordability also has clear potential within a framework of pulmonary rehabilitation to promote exercise engagement and expose patients to “real-life” environments in which to overcome fearful breathlessness safely.

## Conclusions

Using a multi-sensory, fully immersive, VR cycling environment we have shown that perceived breathlessness can be driven independently from physical work effort. This finding highlights the importance of expectation in breathlessness perception which have potential implications for training and rehabilitation programmes.

## Supporting information

S1 File (DOCX)
