# Supplementary material for: Breathlessness in a virtual world: An experimental paradigm testing how discrepancy between VR visual gradients and pedal resistance during stationary cycling affects breathlessness perception
Source: PLoS One. 2023 Apr 21;18(4):e0270721. doi: 10.1371/journal.pone.0270721 (PMC10120935; doi:10.1371/journal.pone.0270721)
Supplement: S1 File — (DOCX) [file pone.0270721.s001.docx]

# Supplementary information

## Generation of the virtual environment

The package contains a single prefab, which includes all the functionality of the system and can be added to a Unity terrain object. Terrains can be specifically constructed or existing ones used, although there are a few caveats such as the immediate road area has to be level. At the beginning of each run, the terrain is altered to match the slope values in the config and all objects are appropriately placed (see fig s1). Gradients are created by increasing/decreasing the height of each terrain height map unit (the absolute dimension of the unit depending on the terrain size/resolution) by the appropriate amount to generate the specified gradient. To create a gentle easing into/out of the slope, there was a gradual increase/decrease in height (1/5 of the normal amount) for the first/last 5 units.

The ANT+ protocol was used to connect to the turbo trainer and set its resistance as well as get the measurement of power generated by the user. To decouple the observed slope from the effort required by the user, the resistance of the turbo trainer was set according to simulated_slope value from the config if the congruent value was false. Fig S2 shows the how the decoupling was achieved and values used for calculating the speed of the virtual bike. A raycast calculation was used to constantly measure the observed slope on the terrain from the cyclist’s perspective, which in most cases is equal to the slope parameter from the config. However, at the end and beginning of each stage, where the slope is easing, the value will be slightly different. This measurement was used to rotate the virtual bike. It was also used in the gravity component of the bike’s movement if congruent was true.

The VR system enables Oculus or steam VR compatible head-sets to be used. VR controllers are used to adjust the position so the VR camera matches that of the virtual cyclist’s head.

***Supplementary Table 1****.* ***Cycling block design.*** *Virtual slope gradient describes what the participant could see (Expectation) and slope resistance describes the resistance applied by the turbo trainer (actual work; e.g., 6% representing the resistance expected when cycling up a 6% gradient). The ‘work’ blocks included for final analyses are highlighted in grey*

| **Block** | **Distance (m)** | **Virtual Slope Gradient (%)**  **‘Expectation’** | **Slope Gradient (%)**  **‘Actual work’** |
| --- | --- | --- | --- |
| **1** | **100** | **0** | **2** |
| **2** | **100** | **0** | **4** |
| **3** | **100** | **0** | **6** |
| **4** | **30** | **0** | **1** |
| **5** | **100** | **4** | **6** |
| **6** | **30** | **0** | **1** |
| **7** | **100** | **8** | **6** |
| **8** | **30** | **0** | **1** |
| **9** | **100** | **6** | **4** |
| **10** | **30** | **0** | **1** |
| **11** | **100** | **10** | **6** |
| **12** | **30** | **0** | **1** |
| **13** | **100** | **4** | **4** |
| **14** | **30** | **0** | **1** |
| **15** | **100** | **12** | **4** |
| **16** | **30** | **0** | **1** |
| **17** | **100** | **2** | **6** |
| **18** | **30** | **0** | **1** |
| **19** | **100** | **10** | **4** |
| **20** | **30** | **0** | **1** |
| **21** | **100** | **6** | **6** |
| **22** | **30** | **0** | **1** |
| **23** | **100** | **12** | **6** |
| **24** | **30** | **0** | **1** |
| **25** | **100** | **2** | **4** |
| **26** | **30** | **0** | **1** |
| **27** | **100** | **8** | **4** |
| **28** | **30** | **0** | **1** |

**Supplementary Table 2. Model summaries for the association between slope resistance and power**

| **ANOVA: Association between slope resistance and power** | | | | |  |
| --- | --- | --- | --- | --- | --- |
|  | *Df* | *Sum Sq* | *Mean Sq* | *F value* | *Pr(>F)* |
| Physical effort of pedalling | 1 | 30984 | 30984 | 30.069 | <0.001 |
| Virtual reality hill gradient | 1 | 2908 | 2908 | 2.822 | 0.0943 |
| Interaction | 1 | 16 | 16 | 0.016 | 0.901 |
| Residuals | 245 | 252452 | 1030 |  |  |

**Supplementary Table 3. Model summaries for predictors of breathlessness**

| **Full mixed effects regression model 1: Predictors of breathlessness** | | | | |
| --- | --- | --- | --- | --- |
| **AIC:** 986.1 | | | | |
|  | *Effect estimate* | *Std. error* | *t-value* | *p-value* |
| Intercept | 4.6982 | 0.3864 | 12.160 | <0.001 |
| Physical effort of pedalling | 0.8623 | 0.2209 | 3.618 | <0.001 |
| Virtual reality hill gradient | 0.6343 | 0.1060 | 5.981 | <0.001 |
| STAI | 0.3596 | 0.2884 | 1.247 | >0.05 |
| BMI | -0.7306 | 0.2735 | -2.671 | 0.02 |
| Age | 0.2969 | 0.2826 | 1.050 | >0.05 |
| Sex (male) | 0.5807 | 0.6080 | 0.955 | >0.05 |

| **Eliminated mixed effects regression model: Predictors of breathlessness** | | | |  |
| --- | --- | --- | --- | --- |
| **AIC:** 366.1 | | | | |
|  | *Effect estimate* | *Std. error* | *t-value* | *p-value* |
| Intercept | 4.9845 | 0.2684 | 18.573 | <0.001 |
| Physical effort of pedalling | 0.8073 | 0.2094 | 3.855 | <0.001 |
| Virtual reality hill gradient | 0.6337 | 0.1058 | 5.987 | <0.004 |
| BMI | -0.7215 | 0.2821 | -2.558 | 0.02 |

**Supplementary Table 4. Model summaries for predictors of physical effort of cycling**

| **Full mixed effects regression model: Predictors of physical effort of cycling** | | | | |
| --- | --- | --- | --- | --- |
| **AIC:** 364.5 | | | | |
|  | *Effect estimate* | *Std. error* | *t-value* | *p-value* |
| Intercept | -0.07967 | 0.14966 | -0.532 | >0.05 |
| Virtual reality hill gradient | 0.09464 | 0.02927 | 3.233 | 0.001 |
| BMI | 0.32592 | 0.10679 | 3.052 | 0.007 |
| Age | -0.22778 | 0.1147 | -1.993 | 0.06 |
| Sex (male) | 0.45171 | 0.22006 | 2.053 | 0.05 |

| **Eliminated mixed effects regression model: Predictors of physical effort of cycling** | | | |  |
| --- | --- | --- | --- | --- |
| **AIC:** 366.1 | | | | |
|  | *Effect estimate* | *Std. error* | *t-value* | *p-value* |
| Intercept | -0.15611 | 0.15985 | -0.977 | >0.05 |
| Virtual reality hill gradient | 0.09467 | 0.02927 | 3.234 | 0.001 |
| BMI | 0.37867 | 0.11432 | 3.312 | 0.004 |
| Sex (male) | 0.60779 | 0.22725 | 2.675 | 0.02 |
